# Paraneoplastic Syndrome After Kidney Transplantation: Frequency, Risk Factors, Differences to Paraneoplastic Occurrence of Glomerulonephritis in the Native Kidney, and Implications on Long-Term Kidney Graft Function

**DOI:** 10.3389/ti.2024.12969

**Published:** 2024-07-25

**Authors:** Izabela Zakrocka, Gayatri Nair, Maria Jose Soler, Kenar D. Jhaveri, Andreas Kronbichler

**Affiliations:** ^1^ Department of Nephrology, Medical University of Lublin, Lublin, Poland; ^2^ Northwell Health, New York, NY, United States; ^3^ Division of Kidney Diseases and Hypertension, Donald and Barbara Zucker School of Medicine at Hofstra/Northwell, Glomerular Center at Northwell Health, New York, NY, United States; ^4^ Nephrology Department, Vall d’Hebron University Hospital, Universitat Autònoma de Barcelona, Barcelona, Spain; ^5^ Nephrology Research Group, Vall d’Hebron Research Institute (VHIR), Barcelona, Spain; ^6^ Department of Internal Medicine IV, Nephrology and Hypertension, Medical University Innsbruck, Innsbruck, Austria

**Keywords:** kidney, transplantation, cancer, paraneoplastic syndrome, glomerulonephritis

## Abstract

Posttransplant malignancies are an important complication of solid organ transplantation. Kidney transplant recipients are at particularly high risk of cancer development. The most relevant risk factors of carcinogenesis are the use of immunosuppressive agents and oncogenic viral infections. Additionally, immune dysregulation caused by these factors may predispose to various types of organ damage. Paraneoplastic glomerular diseases are one of the most interesting and understudied cancer manifestations. The appropriate diagnosis of paraneoplastic glomerular damage can be challenging in kidney transplant recipients, due to factors inherent to concomitant medication and common comorbidities. Recent advances in the field of molecular and clinical nephrology led to a significant improvement in our understanding of glomerular diseases and their more targeted treatment. On the other hand, introduction of novel anticancer drugs tremendously increased patients’ survival, at the cost of kidney-related side effects. Our review aims to provide insights into diagnosis and treatment of paraneoplastic glomerular diseases, with a special attention to kidney transplant recipients.

## Graphical Abstract

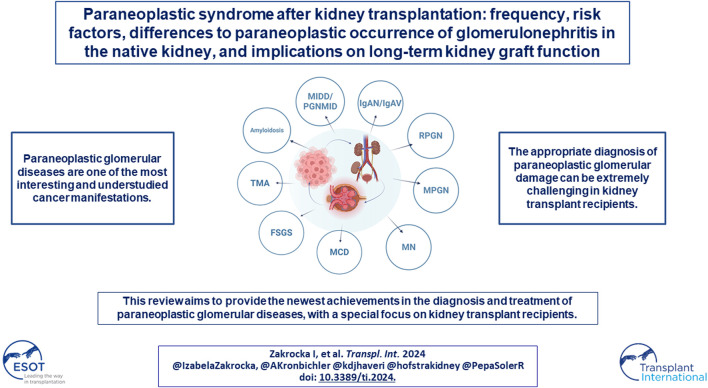

## Introduction

Kidney transplantation is the preferred form of kidney replacement therapy as it offers significant improvement in quality of life and overall survival of patients with end stage kidney disease (ESKD) [1]. Introduction of novel immunosuppressive agents, together with advances in organ preservation and surgical techniques lead to greater 1-year allograft survival [2, 3]. Unfortunately, the rate of long-term complications remains high, reducing overall survival of transplant recipients in comparison to the general population. A plethora of immune- and nonimmune-related factors are known to contribute to late posttransplant complications, with malignancies being one of the most relevant one.

Diagnosis and management of malignancies pose specific challenges in transplant patients. A non-specific presentation of cancer mimicking chronic infectious complications, limited availability of anticancer therapies due to potential nephrotoxic side effects and interactions with immunosuppression are among the main challenges in kidney transplant recipient. Identified risk factors of posttransplant malignancies are older age at the time of transplantation, male gender, white ethnicity, time spent on dialysis, longer term follow-up after transplantation, and higher cumulative exposure to immunosuppression [4]. Malignancies are reported as the second most common cause of death in kidney allograft recipients and the overall cancer risk is estimated to be 2- to 4- fold higher than in the general population [5].

Paraneoplastic syndromes, defined as cancer clinical manifestations triggered by tumor products, but not by the neoplasm itself, may precede cancer diagnosis or occur even many years thereafter [6]. The diagnostic processes of paraneoplastic syndrome in kidney transplant patients can be difficult due to its non-characteristic clinical picture and often require invasive procedures to make a final diagnosis. One of such paraneoplastic syndromes are glomerular diseases. Appropriate diagnosis is of special importance, since glomerular diseases in kidney transplant recipients are claimed to be the second most common cause of kidney graft failure [7]. Among possible triggers of glomerular damage after kidney transplantation, a complex interplay between genetic predisposition and especially immunological factors seems to play a leading role. Three types of glomerular damage can occur: donor-derived, *de novo* and recurrent. *De novo* glomerulonephritis is mainly immune complex mediated, being highly dependent on the immune status of the graft recipient [8]. In contrast, recurrent glomerular diseases are mainly found in kidney transplant recipients with an established glomerular disease before transplantation, often resulting in significant graft function impairment and eventually its loss [9]. Adding to that, a specific type of glomerular damage, classified as paraneoplastic glomerular disease, remains intriguing and an understudied kidney allograft complication. Although one of the first reports connecting the presence of nephrotic syndrome with malignant tumors was already published in 1966 [10], data about paraneoplastic glomerular diseases in kidney transplant patients remain obscure.

In this review, we aim to describe the epidemiology of paraneoplastic glomerular diseases after kidney transplantation, differences in their management in comparison with non-transplant patients, their impact on graft survival and difficulties regarding immunosuppressive and anticancer treatment. The paucity of data in the posttransplant setting requires in-depth discussions of paraneoplastic glomerular diseases in the native kidney.

## Paraneoplastic Glomerular Diseases

Paraneoplastic glomerular diseases are defined as disorders that are not attributable to invasion or compression by the tumor or by metastasis caused by the tumor [11]. The production and secretion of hormones or peptides by the tumor or immune cross-reactivity with the host’s renal tissue are considered to lead to paraneoplastic glomerular diseases. A paraneoplastic origin is likely when the occurrence of glomerular disease has a temporal relationship to the occurrence of cancer and the severity of kidney disease follows the course of cancer, i.e., deteriorates when the tumor burden increases or *vice versa*. This has been nicely demonstrated for a case with thrombospondin type-1 domain-containing 7A (THSD7A)-associated membranous nephropathy (MN) who had a concomitant gallbladder carcinoma. After surgery of the carcinoma, THSD7A antibodies became negative, and a subsequent significant reduction of proteinuria indicated a partial response of MN to anti-cancer measures [12].

## Risk Factors of Paraneoplastic Glomerular Diseases After Kidney Transplantation

### Drugs

**FIGURE 1:**
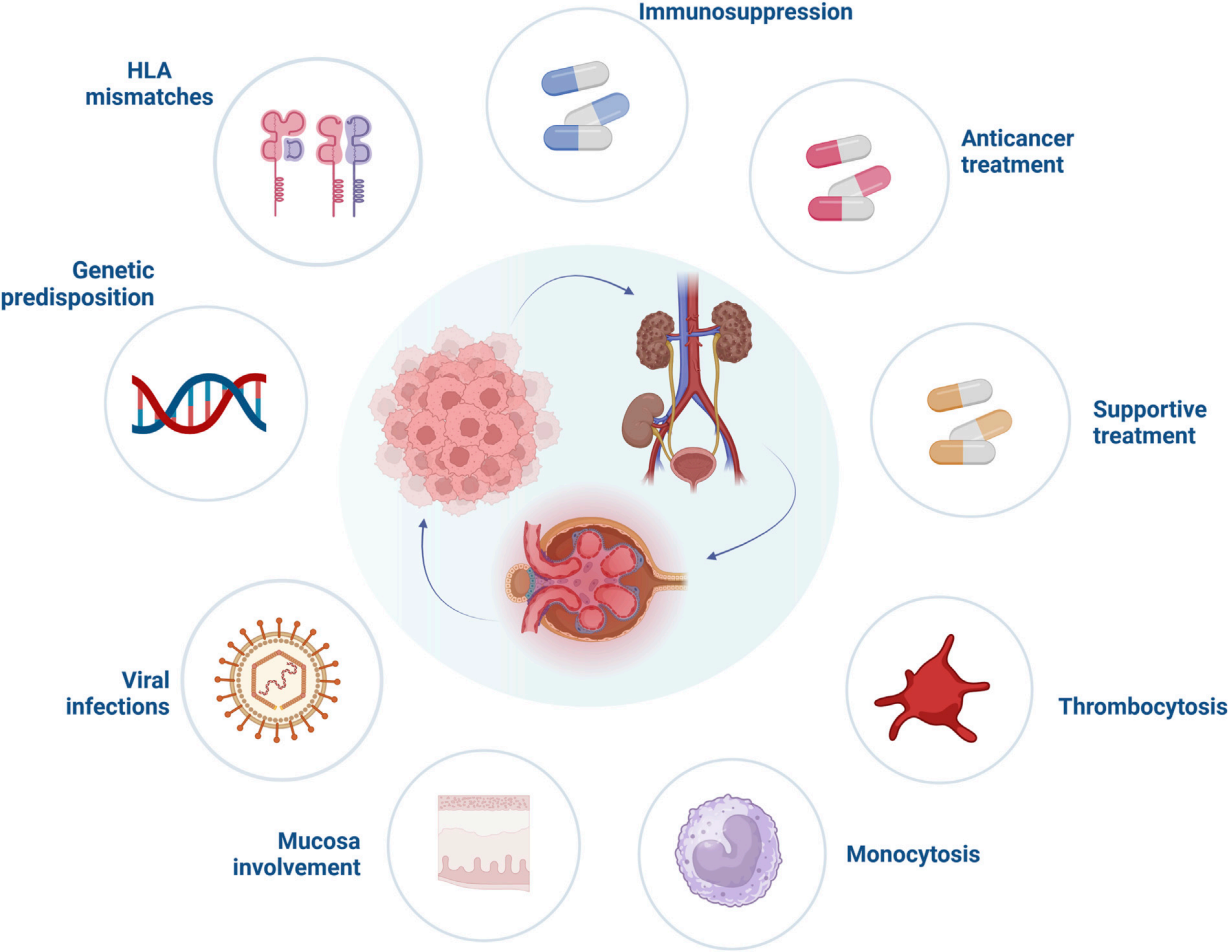
Risk factors involved in the pathogenesis of paraneoplastic glomerular diseases. Abbreviations: HLA, human leucocyte antigen. Created with BioRender.com.

Little is known whether one specific agent is responsible for an increased frequency of paraneoplastic glomerular disease (Figure 1). Alemtuzumab, a monoclonal antibody directed against the CD52 antigen, is known to cause autoimmunity after its use. Post-marketing surveillance in patients with multiple sclerosis found that one out of three will develop another autoimmune disorder after alemtuzumab is used, but these cases are mainly restricted to thyroid dysfunction and only a minority (<10%) thereof are considered serious [13]. Nonetheless, severe autoimmunity such as the development of anti-glomerular basement membrane (GBM) disease has been reported in single cases [14]. Associations between administration of other potent induction immunosuppressants and systemic autoimmunity are much weaker, but single cases of Guillain-Barre syndrome with the use of anti-thymocyte globulin exist in the literature [15]. It should be noted, that induction therapy itself with antithymocyte globulin or alemtuzumab significantly increases the risk of malignancy development, especially posttransplant lymphoproliferative disorder [16]. Taken together, a paucity of data on the prevalence of paraneoplastic glomerular disease development with the use of agents to prevent allograft rejections hinders strong conclusions, but it is likely that certain agents such as alemtuzumab at least influence the occurrence of glomerular diseases independent of cancer development.

### HLA Matching

Besides the possible role of immunosuppression in paraneoplastic glomerular diseases pathogenesis, other specific factors related to solid organ transplantation should be mentioned (Figure 1). It is widely accepted that human leukocyte antigen (HLA) matching is essential for optimal graft function, however, it is not required for cancer prevention. Khurram et al. inoculated rats with kidney tumor cells and started them on cyclosporine. The animals showed stronger anti-tumor response when a higher degree of major histocompatibility complex (MHC) mismatch was present in comparison to a well-matched group [17]. Moreover, after withdrawal of immunosuppression all rats in the mismatch group eliminated cancer cells, compared to only a 50% reduction in well-matched animals, suggesting a significant impact of immunosuppression reduction and higher mismatch levels on cancer prognosis. Unfortunately, data from human studies are less conclusive. In a large study analyzing data from 166,256 adult kidney transplant recipients, who survived 1 year without episodes of graft rejection or malignancy, recipients with 4–6 HLA mismatches had higher risk of solid organ cancer development (hazard ratio [HR] = 1.11, 95% CI = 1.00–1.34) compared to well-matched individuals. This, however, might have also been related to other risk factors, such as male sex or prior transplant history [18]. On the contrary, an analysis focusing on the incidence and risk factors of melanoma in a cohort of 105,174 kidney transplant recipients from the United States Renal Data System database observed that less than 4 HLA mismatches significantly increased the risk of melanoma development (44.9% vs. 37.1%) [19]. This finding was supported by another study performed on 10,649 heart and lung transplant recipients, which indicated that the higher level of HLA mismatching revealed a protective effect on occurrence of posttransplant skin cancers [20]. However, little is known whether higher levels of HLA mismatches provide protection against recurrence or *de novo* occurrence of glomerular disease. Fewer HLA mismatches have been postulated as a potential risk factor of glomerulonephritis reoccurrence, particularly IgA nephropathy [21]. The significance of HLA mismatch in the pathogenesis of paraneoplastic glomerular diseases remain to be established by future studies.

### T cell Subpopulations

A study in kidney transplant recipients with cancer found a significantly higher frequency of Tregs together with higher level of soluble HLA-G (sHLA-G) [22]. Tregs have been shown to exert immunosuppressive features, thereby potentially preventing the occurrence of paraneoplastic autoimmunity [23]. A subsequent reduction of Treg activity, eventually developing an exhaustive phenotype, may thereby provoke specific glomerular damage as well.

Beyond the crucial role of specific T cells on alloantigen recognition and antibody production, other cells, i.e., natural killer (NK) cells exhibit alloreactive properties, relevant to control viral infections after kidney transplantation, such as cytomegalovirus (CMV) or Ebstein-Barr (EBV) infections [24]. This alloreactivity may in turn result in glomerular damage. Xiao et al. reported a case of histiocytic glomerulopathy related with extranodal NK/T-cell lymphoma [25]. On the other hand, NK cells have been postulated as potential biomarkers of idiopathic MN, their levels significantly increased after rituximab treatment [26] and were associated with remission of MN [27]. In a recently published study, antigen-specific chimeric autoantibody receptor (CAAR) NK cells have been used to eliminate cells producing antibodies involved in MN, especially against THSD7A [28]. The specific role of immune cells in the development of paraneoplastic glomerulopathies requires further studies.

### Viruses

Viral infections are another important factor related to impaired graft function and cancerogenesis in kidney transplant recipients (Figure 1). In addition, it was suggested that viruses may induce glomerular damage. One of these, Kaposi’s sarcoma herpesvirus (KSHV)/human herpesvirus 8 (HHV8) has high tropism for endothelial cells, thereby inducing vIL-6 [29] and vascular endothelial growth factor (VEGF) [30] as angiogenic factors. vIL-6 has been proposed as one of the molecular links between KSHV and glomerular diseases, especially amyloidosis, thrombotic microangiopathy (TMA) and membranoproliferative glomerulonephritis (MPGN) [31]. Interestingly, KSHV gene sequences have been found in patients with multiple myeloma and primary amyloidosis [32]. Other viral infections may contribute to glomerular damage after kidney transplantation as well. Cases of immunotactoid [33] and crescentic glomerulopathy [34] secondary to CMV infection have been documented.

### Genes

Underlying genetic predisposition is of relevance for paraneoplastic glomerular diseases. Mutations of more than 70 genes are already identified to be associated with increased risk of glomerular damage, however often a “second hit” is necessary to evoke the full clinical picture of kidney disease, frequently manifesting as focal segmental glomerulosclerosis (FSGS), a non-specific pattern of kidney injury, in a kidney biopsy [35]. Bonilla et al. suggested that mutations of genes crucial for podocyte function, namely, protocadherin FAT1 gene, may be one of the unrecognized triggers of immune-mediated glomerular damage, especially in the transplant setting [36]. Moreover, mutations of podocyte genes may predispose to glomerulopathies secondary to anticancer treatment. Czogalla et al. presented another “multi-hit” case of FSGS in a patient with chronic lymphocytic leukemia, in whom an unrecognized podocin mutation was exacerbated by ibrutinib administration [37]. Additionally, a specific role of genotyping the donor has been highlighted in other studies. This is particularly relevant in donors of African-American decent, where apolipoprotein L1 (APOL1) gene polymorphisms play a crucial role, and a risk constellation (G1/G2) is related to collapsing FSGS in kidney transplant recipients, which in turn might rapidly progress to ESKD [38].

### Complement System

The role of complement system activation in cancerogenesis remains a controversial issue. It was postulated that local complement activation in the microenvironment of melanoma is responsible for lower immune responsiveness to neoplastic cells through inhibition of CD8^+^ T cells [39]. On the other hand, reduced C3 and C5 production or the blockade of the C5 receptor reduced tumor growth in animal models of cancer, indicating their crucial role in cancerogenesis [40]. Cancer or treatment-related complement activation may also result in TMA, a rare form of glomerular damage, and is in some cases more amenable to anticomplement treatment than therapeutic plasma exchange (TPE) [41].

## Types of Paraneoplastic Glomerular Diseases

**FIGURE 2:**
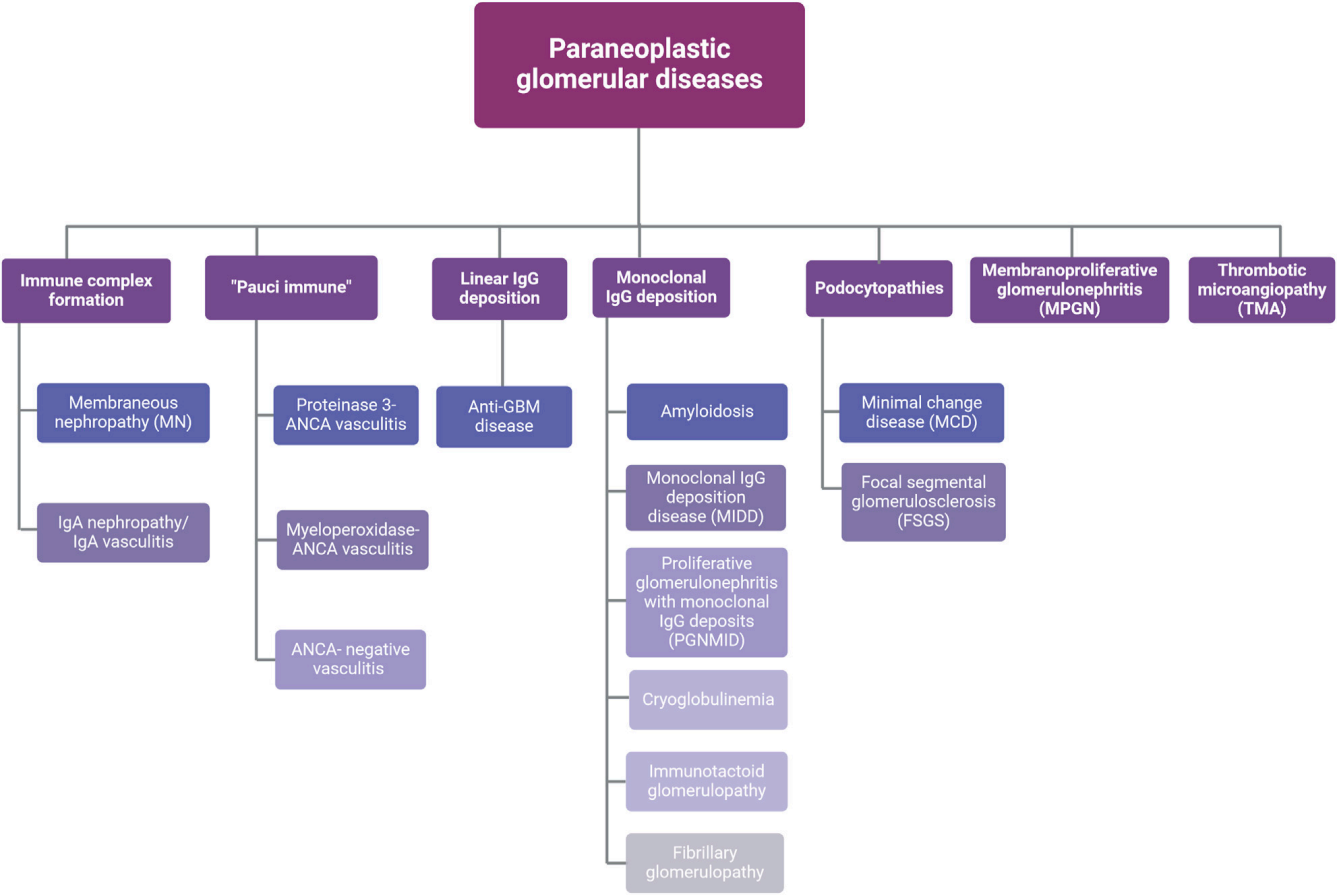
Types of paraneoplastic glomerular diseases. Abbreviations: ANCA, anti-neutrophil cytoplasmic antibodies; GBM, glomerular basement membrane; IgA, immunoglobulin A; IgG, immunoglobulin G. Created with BioRender.com.

Several patterns of glomerular injury have been linked with cancer occurrence (Figure 2). In the next sections the most common histological types of paraneoplastic glomerular diseases will be discussed.

### Membranous Nephropathy (MN)

MN is the most common type of paraneoplastic glomerular disease related to solid tumors and is less prevalent in hematological malignancies. The prevalence of paraneoplastic cases of MN was reported to be approximately 20% [42]. Importantly, the diagnosis of MN may precede the occurrence of malignancy [43]. Gastrointestinal cancers, followed by lung, kidney and prostate cancer are the most frequently reported malignancies to cause MN [6]. MN usually manifests as nephrotic syndrome, with formation of immune complexes leading to its onset. However, it should be noted that although glomerular immune deposits were found in the autopsy of 30% of cancer patients, their significance remains unclear [44]. A higher deposition of IgG1 and IgG2 was found in patients with paraneoplastic MN [45], whereas IgG4 prevalence was related with the primary form of MN [42]. Additionally, a switch in the IgG subclass was shown to predict MN progression, especially in secondary MN [46]. Other findings, such as >8 inflammatory cells affecting the glomerulus, mesangial proliferation and segmental MN, especially in the presence of neural epidermal growth factor-like 1 (NELL-1) antigen, support the diagnosis of paraneoplastic MN [47]. The discovery of novel antigens involved in MN pathogenesis significantly improved our diagnostic understanding of glomerular damage in these patients. Antibodies against the M-type phospholipase A2 receptor (PLA2R) are known to be highly indicative for primary MN. Recently discovered antigens, i.e., THSD7A, FAT1, NELL-1 and protocadherin 7 (PCDH7) have been postulated as potential antigens in paraneoplastic MN [48]. Importantly, the diagnosis of PLA2R positive MN does not exclude paraneoplastic occurrence of MN, since reports of cases with positive PLA2R staining are available [49]. Moreover, dual antigen-positive MN cases have been reported, with predominant IgG1 staining in the kidney tissue and clinically a longer time to achieve remission [50]. Despite great advances in diagnostic techniques, the course of MN in kidney transplant recipients remains understudied. Solà-Porta et al. reported an unusual case of a 72-year old kidney transplant patient with THSD7A-positive MN and positive C4d capillary wall staining, without anti-THSD7A antibodies and lesions suggestive for malignancy [51]. In another study, Münch et al. presented a case of NELL-1-positive MN in a 56-year old kidney transplant recipient, again without underlying malignancy, in whom the level of serum anti-NELL-1 antibodies correlated with the proteinuria intensity [52]. Since NELL-1 was found in noncancerous MN cases, i.e., after exposure to lipoic acid [53], tiopronin [54] or mercury [55], more data are needed to predict the role of MN antigens on the kidney graft function and outcome, especially in the presence of malignancy.

### Minimal Change Disease (MCD)

**FIGURE 3:**
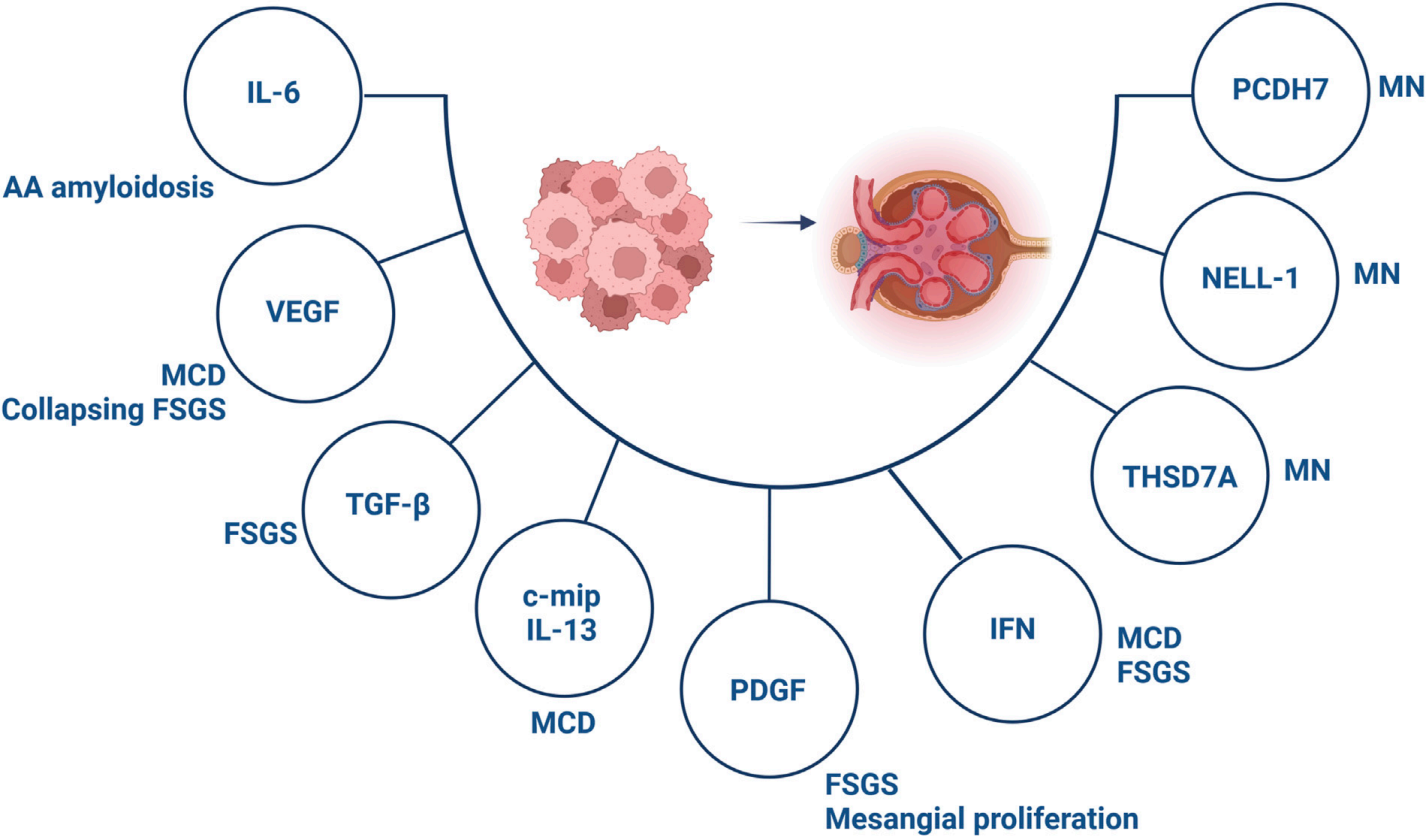
Involved components in the pathogenesis of paraneoplastic glomerular diseases. Abbreviations: c-mip, c-maf inducing protein; FSGS, focal segmental glomerulosclerosis; IFN, interferon; IL, interleukin; MCD, minimal change disease; MN, membranous nephropathy; NELL-1, neural epidermal growth factor-like 1; PCDH7, protocadherin 7; PDGF, platelet-derived growth factor; TGF-β, transforming growth factor-β; THSD7A, thrombospondin type 1 domain containing 7A; VEGF, vascular endothelial growth factor. Created with BioRender.com.

Hematological malignancies, especially Hodgkin’s lymphoma, non-Hodgkin’s lymphoma and leukemias have often been associated with MCD, although single cases of solid tumors affecting gastrointestinal tract, lung, kidney and thymus have been described [56]. The role of cytokines secreted by cancer cells is of special importance in the pathogenesis of paraneoplastic MCD (Figure 3).

Nephrotic syndrome evoked by paraneoplastic MCD occurs early, around the time of malignancy diagnosis. High levels of circulating cytokines released by lymphocytes and macrophages are responsible for a generalized inflammatory condition, resulting in systemic symptoms. Paraneoplastic MCD was reported to remit after successful anti-cancer treatment, resulting in either complete [57, 58] or partial remission [59]. MCD occurs frequently after hematopoietic stem cell transplantation, likely as a limited form of graft *versus* host disease manifesting in the kidney [60]. Data on MCD occurrence after kidney transplantation are scarce. Yamada et al. reported a case of a patient 25 years after transplantation, who presented with nephrotic syndrome due to minimal change like podocyte injury evoked by coronavirus disease-2019 infection and with high-risk APOL1 alleles in the kidney donor, that improved after glucocorticoid administration [61]. Another case of a combined heart-kidney transplant recipient developing *de novo* MCD more than 1 year after transplantation was reported who responded to glucocorticoids [62].

### Immunoglobulin a (IgA) Nephropathy

The association between IgA nephropathy and malignancies has first been reported in 1984 [63]. Among the most common cancers, respiratory tract and buccal cavity cancers have been reported [6], although rare cases of hematological malignancies are published [64, 65]. IgA nephropathy has emerged as the most common glomerular disease when kidney biopsies were performed in cancer patients, with a frequency of 7.4%, followed by MN in 6.1% [66]. The time of paraneoplastic glomerulopathy manifestation can also differ in various cancers. In a case presented by Melandro et al., the diagnosis of IgA nephropathy preceded kidney graft cancer occurrence by 6 months [67]. Interestingly, mesangial IgA deposits have been found in the autopsy of 17% of patients with mainly gastrointestinal malignancies, and without prior evidence of kidney damage [68]. Tumor antigens and abnormal IgA production by cancer cells, together with abnormal immune system reactivity against tumor antigens have been suggested to be involved in paraneoplastic IgA nephropathy [69]. Tumor removal remains the standard therapy in cancer-related IgA nephropathy [70].

### Anti-Neutrophil Cytoplasmic Antibodies (ANCA)-Associated Vasculitis

Patients with anti-neutrophil cytoplasmic antibody (ANCA)-associated vasculitis are at higher risk to develop malignancies, with frequencies reported from 10% to 26%, with a significant increase 5 years after diagnosis [71]. These estimates largely stem from the cyclophosphamide- and azathioprine-era, both known to be carcinogenic. Recent investigations have found a standardized incidence ratio which is comparable to a background population [72]. Cyclophosphamide is still used by many practicing centers in the management of severe kidney failure due to ANCA-associated vasculitis. This was linked to increased frequency of several malignancies, including prostate [73], breast [74], respiratory tract [75] and chronic lymphocytic leukemia [76]. Removal of malignancy is not sufficient to control active vasculitis, as has been highlighted in a case report where an intensive immunosuppressive treatment including TPE was shown to be effective in a 85-year old patient with prostate cancer and ANCA-negative pauci-immune glomerulonephritis [77].

### Anti-Glomerular Basement Membrane (Anti-GBM) Disease

Anti-GBM disease is a rare but severe kidney disease, frequently presenting with dialysis-dependent kidney failure. Due to the severity of symptoms, immediate care is required to optimize renal and overall outcome of patients. Paraneoplastic anti-GBM disease has been reported in patients with renal cell carcinoma [78, 79], lung [80] and rectal cancer [81]. Although different therapeutic approaches were shown, immunosuppression alongside TPE was effective in achieving partial remission of paraneoplastic anti-GBM disease [82].

### Focal Segmental Glomerulosclerosis (FSGS)

FSGS describes a lesion encountered by different triggers. The pathogenesis of paraneoplastic FSGS is, in line with MCD, based on VEGF expression and secretion of profibrotic factors (Figure 3). Several malignancies have been linked to FSGS, although mainly of hematological origin [83, 84]. Importantly, essential thrombocythemia and polycythemia have been associated with FSGS, which has been assumed to occur secondary to cytokine release responsible for glomerulosclerosis [85, 86]. Data on the effectiveness of anticancer treatment and concomitant immunosuppression in paraneoplastic FSGS remain elusive, indicating achievement of FSGS remission in some cases [87].

### Thrombotic Microangiopathy (TMA)

TMA is another rare presentation of paraneoplastic glomerular injury. The correlation between the TMA and cancers has been shown in mucin-producing neoplasms, especially stomach, breast and lung [88]. Patients are exposed to TMA risk factors after kidney transplantation [89], and most *de novo* cases were observed in the graft [90, 91]. Of note, paraneoplastic TMA diagnosis after kidney transplantation remains a huge challenge, since its histological picture often mimics antibody-mediated or T-cell mediated rejection [92, 93]. However, lack of laboratory indicators of generalized intravascular thrombosis, *de novo* TMA localized in the graft, lack of donor specific antibodies (DSAs) and/or C4d deposition in peritubular capillaries and time between occurrence of cancer and TMA may help to distinguish primary and posttransplant TMA from its paraneoplastic form. Treatment of paraneoplastic TMA is also a matter of debate. It has been reported that TPE is less effective in cancer-derived TMA, due to lower rate of reduced ADAMTS13 activity [88]. Additionally, single reports emerged about the effectiveness of eculizumab in cancer-associated TMA [94]. More studies are necessary to analyze the pathogenesis of paraneoplastic TMA and to expand the armamentarium of therapies that can be safely used, especially in kidney transplant recipients.

## Newer Anticancer Drugs and Glomerular Damage

Onconephrology, and in particular transplant medicine are rapidly evolving fields, providing targeted treatment in kidney transplant recipients diagnosed with cancer. Up to date, various anticancer agents have been shown to induce glomerular damage (Figure 1) [36]. Novel anticancer drugs have revolutionized oncological treatment and significantly improved patient survival. Immune checkpoint inhibitors (ICIs) block cytotoxic T-lymphocyte antigen 4 (CTLA-4), programmed cell death 1 receptor (PD-1) or its ligand (PD-L1), main targets responsible for downregulation of T cells. As a result, an anticancer response after ICIs is augmented, at the cost of immune-mediated events [95]. A broad spectrum of glomerular disorders has been linked with ICIs use, with both nephrotic and nephritic presentation. In a systematic review performed by Kitchlu et al. the most common form of glomerular damage found after ICIs administration was pauci-immune glomerulonephritis, followed by MCD, C3 glomerulonephritis, amyloid A amyloidosis and IgA nephropathy [96]. Most patients presenting with glomerular lesions related to ICIs have been successfully treated with corticosteroids or a combination of rituximab or cyclophosphamide and corticosteroids (the latter especially in cases with pauci-immune glomerulonephritis) [97], nonetheless, critical questions remain largely unanswered. First, reintroduction of ICIs is challenging, especially when pauci-immune glomerulonephritis was induced by ICIs, as of recurrence of glomerular diseases might be expected. Second, patients after kidney transplantation are at high risk of immune-mediated disorders, especially due to immunotherapy. In general, kidney transplant recipients treated with ICIs experience more often episodes of antibody mediated as well as T cell mediated rejections. Increasing the dose of corticosteroids and switching calcineurin inhibitors (CNIs) to mammalian target of rapamycin (mTOR) inhibitors prior to ICIs initiation are one of the proposed resolutions to reduce the risk of immune-related episodes of graft injury in kidney transplant recipients [98]. However, one needs to emphasize that mTOR inhibitors may induce glomerular damage in the form of TMA or FSGS, probably due to overexpression of VEGF in podocytes [56].

Beyond anticancer drugs and their potential to induce glomerular damage, the role of supportive treatment of glomerulopathies should be mentioned (Figure 1). Potent stimulators of granulocyte and macrophage activity, granulocyte-colony stimulating factors filgrastim and its derivative pegfilgrastim were associated with glomerular damage in patients with hematological malignancies [56, 99, 100].

## Paraneoplastic Glomerular Disease and Implications on Long-Term Graft Function in Kidney Transplant Recipients

The immunological risk related to conversion of immunosuppression at the time of cancer diagnosis in kidney transplant recipients is challenging. Lack of recommendations regarding the management of immunosuppression in cancer patients after organ transplantation enforces clinicians to balance between the risk of graft rejection (also due to paraneoplastic glomerulopathy) and effective anticancer treatment [101]. Owing to limited effectiveness of standard treatment (immunosuppression, TPE) of paraneoplastic glomerular diseases, which mostly depends on cancer removal and adequate anticancer pharmacotherapy, long-term graft function preservation can be challenging, especially in kidney transplant recipients.

## Conclusion

To the best of our knowledge, this is the first comprehensive narrative review of paraneoplastic glomerular diseases, with special focus on patients after kidney transplantation. Our article highlights further areas of research, as such cases are underreported and in clinical routine pose challenges for the treating physicians. Close cooperation between nephrologists, specialists in transplantation and hemato-oncologists should ensure appropriate medical care of kidney transplant recipients diagnosed with cancer. Large inter-center studies are crucial to determine the significance of paraneoplastic glomerular diseases after kidney transplantation.

## Figures and Tables

**Table:** 

**ANCA**	Anti-neutrophil cytoplasmic antibody
**APOL1**	Apolipoprotein L1
**CAAR**	Chimeric autoantibody receptor
**c-mip**	c-maf inducing protein
**CMV**	Cytomegalovirus
**CNI**	Calcineurin inhibitor
**CTLA4**	Cytotoxic T-lymphocyte antigen 4
**DSA**	Donor specific antibody
**EBV**	Ebstein-Barr virus
**ESKD**	End stage kidney disease
**FSGS**	Focal segmental glomerulosclerosis
**GBM**	Glomerular basement membrane
**HHV-8**	Human herpesvirus-8
**HLA**	Human leukocyte antigen
**ICI**	Immune checkpoint inhibitor
**IgA**	Immunoglobulin A
**IL**	Interleukin
**KSHV**	Kaposi’s sarcoma herpesvirus
**MCD**	Minimal change disease
**MHC**	Major histocompatibility complex
**MN**	Membranous nephropathy
**MPGN**	Membranoproliferative glomerulonephritis
**mTOR**	Mammalian target of rapamycin
**NELL-1**	Neural epidermal growth factor-like 1
**NK**	Natural killer
**PCDH7**	Protocadherin 7
**PD-1**	Programmed cell death 1
**PLA2R**	Phospholipase A2 receptor
**sHLA-G**	Soluble HLA-G
**TGF-β**	Transforming growth factor β
**THSD7A**	Thrombospondin type-1 domain-containing 7A
**TMA**	Thrombotic microangiopathy
**TPE**	Therapeutic plasma exchange
**VEGF**	Vascular endothelial growth factor
